# Coexisting Papillary and Clear Renal Cell Carcinoma in the Same Kidney

**DOI:** 10.1155/2014/575181

**Published:** 2014-08-10

**Authors:** Murat Ustuner, Busra Yaprak, Kerem Teke, Seyfettin Ciftci, Mucahit Kart, Kursat Yildiz, Melih Culha

**Affiliations:** ^1^Department of Urology, University of Kocaeli, Campus of Umuttepe, 41380 Kocaeli, Turkey; ^2^Department of Pathology, University of Kocaeli, Kocaeli, Turkey; ^3^Department of Urology, Banaz State Hospital, Usak, Turkey

## Abstract

Renal cell carcinoma (RCC) is the most common solid lesion of the kidney. Bilateral synchronous benign and malignant renal tumors have been defined in some reports. However, unilateral concordance of malignant renal tumors is very rare and there are only a few cases that had synchronous different subtypes of malignant renal tumors arising within the same kidney. Herein, we describe a 67-year-old male patient who had clear cell RCC and papillary RCC in his right kidney that were successfully treated with radical nephrectomy. We also reviewed the pertinent literature.

## 1. Introduction

Renal cell carcinoma (RCC) is the most common solid lesion of the kidney and accounts for approximately 2-3% of all malignancies in adults [1]. The most common subtypes of renal cell carcinoma are clear cell, papillary, and chromophobe type RCC and account for approximately 80%, 10%, and 5%, respectively. Renal oncocytoma, angiomyolipoma (AML), and renal adenomas are the benign tumors of the kidney. There are a few of studies that define bilateral synchronous malignant renal tumors [2–4] or coexisting benign and malignant tumors arising within the same kidney [5]. To the best of our knowledge, clear cell RCC and papillary RCC arising within the same kidney are very rare in the literature. Herein, we describe a case of a 67-year-old male who had 2 different subtypes of renal cell carcinoma in the same kidney that were successfully treated with radical nephrectomy (RN). We also reviewed the related literature.

## 2. Case Report

**Figure 1:**
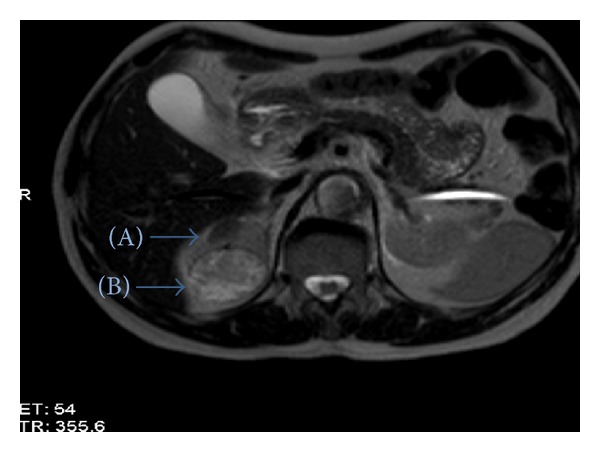
Preoperative MRI image of the patient. Cystic lesion (A) and solid lesion (B).

A 67-year-old man was admitted to another medical center with right flank pain lasting for more than one month. An ultrasound scan revealed a mass in the right kidney. His subsequent computed tomography (CT) scan and magnetic resonance imaging (MRI) revealed a mass on the upper pole of right kidney, 4.5 cm diameter, exophytic and solid character suggestive for malignancy. MRI scan also showed a 2 cm diameter, hypodense mass on the upper pole of right kidney which was thought to be a benign cystic lesion (Figure 1). However this lesion was not revealed at CT. He was referred to our department. Physical examination was normal. All hematological and biochemical tests were unremarkable except total PSA (7.07 ng/dL). Patient had no previous medical history. He was a smoker for 40 years.

The tumor was clinically diagnosed as a right renal tumor and classified as cT1bN0M0, according to tumor-node-metastasis system. Patient underwent right RN and adrenalectomy. He was discharged at postoperative third day, without any complication. At the sixth month and first year follow-up, the patient was free from the disease. 

*Pathology.* On macroscopic examination, there were 2 different tumors on the upper pole of right kidney. The cut surface of the bigger one was at 6.5 cm diameter, composed of two nodules, one of them was of orange color and the other one was of yellow-brown color and had small cystic cavities. The smaller tumor was in 1 cm diameter, of beige color, and had irregular margins. The distance between two tumors was 0.5 cm. Microscopy revealed two different patterns. The bigger tumor was diagnosed as clear cell type RCC with a diameter of 6.5 cm and smaller one was diagnosed as type 1 papillary RCC with a diameter of 1 cm on microscopic examination. Both of the tumors were limited in the kidney and there was no perirenal and perihilar invasion. There was no invasion of blood and lymph vessels. Pathologic examination revealed a normal adrenal gland.

**Figure 2:**
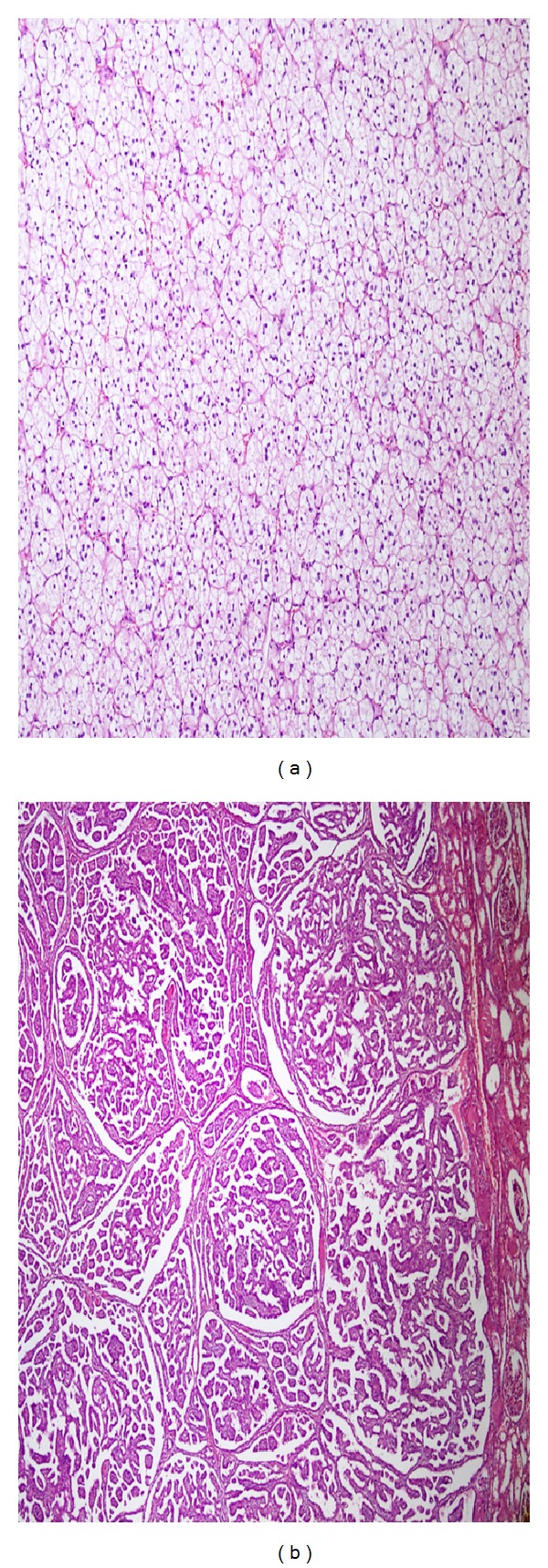
Microscopic images of clear cell RCC (a) and papillary type RCC (b).

Areas of clear cell RCC were characterized by solid nests and sheets of carcinoma cells interspersed by prominent network of delicate blood vessels. Tumor cells have water clear cell cytoplasm surrounded by a distinct cell membrane and had Fuhrman grade 2 morphology with finely granular chromatin but small nucleoli that are not discernible of 10x magnification. On the other hand, areas of papillary RCC were characterized by tightly packed tubulopapillary structures and nuclear morphology was consistent with Fuhrman grade 2 (Figure 2).

## 3. Discussion

RCC comprises 2-3% of all cancers [1]. The incidence of RCC has also risen over the past several decades due to incidental detection [6]. The best known etiological factors for all types of RCC are smoking, obesity, and hypertension [7]. Smoking is clearly implicated in the etiology of RCC, with a strong dose-dependent increase in risk associated with numbers of cigarettes smoked per day and substantial reduction in risk for long-term former smokers. Smoking men who had smoked 1–9, 10–20, or 21 or more cigarettes/day had a relative risk (RR) of 1.60 (95% CI = 1.21–2.12), 1.83 (95% CI = 1.30–2.57), and 2.03 (95% CI = 1.51–2.74), respectively. And the advantages of smoking cessation were confirmed by a reduction in RR for those who had quit smoking for >10 years as compared to those who had quit for 1–10 years [8]. Among the documented etiologic causes that were described above, smoking was the only etiologic factor in our case. There was a 40-year history of smoking and a 2.03 RR of kidney cancer for this case.

In general, renal tumors are in solid or cystic character according to the imaging findings. Surgical excision (partial nephrectomy (PN) or RN) is recommended for all localized RCCs [9]. So, in preoperative assessment, numbers of tumor do not change the excision criteria of renal tumors. However, in pathologic evaluation, different types of tumors can be defined. RCC comprises several subtypes with specific histopathology and genetic characteristics, the most commonly diagnosed including clear cell, papillary, and chromophobe. Histopathologically, clear cell RCC has clear cytoplasm with solid, tubular, or cystic growth pattern. Two different papillary tumor subtypes were defined: type 1 with small cells and pale cytoplasm and type 2 with large cells and eosinophilic cytoplasm. Both clear cell and papillary types of RCC originated from proximal tubules.

There are some literatures that define the synchronous benign and malignant tumors in the same kidney. Specially, AML and RCC have been defined many times in the literature in tuberous sclerosis (TSC) and non-TSC patients. Billings et al. [5] defined an 86-year-old woman without TSC with a coexisting 7 cm clear cell RCC and 9.5 cm AML in the same kidney that were treated with right RN successfully. They also stated pertinent literature of 31 cases that had AML and RCC in same kidney. Among them, 17 had TSC and 14 did not. Khallouk et al. [10] defined a case report of a 35-year-old male with TSC and bilateral massive AML. They performed right RN successfully and pathology revealed AML and clear cell RCC in the same kidney. Besides these, oncocytoma has been defined with malignant tumors arising within the same kidney [11].

In addition to coexisting benign and malignant tumors in the same kidney, there have been described some reports that define the coexisting 2 different types of RCC. Kuroda et al. [12] defined a case of 2 different types of clear cell RCC: clear cell RCC and clear cell papillary type RCC. Clear cell papillary type RCC has been recently defined. The authors reported a case of a 57-year-old male patient with a left renal tumor. They performed left RN and found 3 tumors in the same kidney. Microscopic evaluation revealed 2 different types of clear cell RCC: clear cell and clear cell papillary type RCCs. After the genetic evaluation, they conclude that clear cell papillary type RCC is different from clear cell RCC or papillary RCC. Furthermore, Kang et al. [13] reported a 43-year-old female who underwent left RN with the diagnosis of AML and suspicion of RCC. Pathologic examination revealed multiple AML with chromophobe RCC and clear cell RCC. Beside these studies, Richstone et al. [14] retrospectively analysed 1071 patients who underwent RN due to renal tumors in terms of multifocality. The authors reported 57 (5.3%) cases of multifocality and 6 of them were bilateral synchronous renal tumors. Among these multifocal cases, they found 74% of the cases had same histological subtypes. They also defined that papillary subtype was observed in 11.4% of RCC patients, while in 37% in multifocal group. Thus, they found that papillary subtype RCC was significantly associated with the multifocality. They also reported that 9 of the 57 patients had pathologic discordance between primary and satellite tumors with a clear cell RCC and papillary type RCC. However, they did not clarify any patient that had these different subtypes of tumors within the same kidney. In contrast to their result, the histologic subtypes of our case were different. Similar to their result, papillary type RCC is one of the components of multifocal tumors in our case.

In the literature, we are aware of only 2 articles that reported synchronous clear cell RCC and papillary RCC in the same kidney, similar to our case [15, 16]. Simhan et al. [15] reported the data of 97 patients who had multifocal renal tumors. They reported 8 patients who had mixed (papillary and clear cell) RCC, all of which were treated by PN. Capaccio et al. [16] found 7 patients who had unilateral synchronous tumors with different subtypes. One of them had oncocytoma and one had clear cell RCC with synchronous AML. Remaining 5 patients had different histological subtypes of RCC, 3 of which had synchronous papillary and clear cell type RCC. All of these 3 patients were treated by RN and only one of them died from the disease 5 years after the operation. The other 2 patients had chromophobe subtype RCC with unilateral synchronous papillary type in 1 patient and clear cell type RCC in 1 patient.

In fact, there is not sufficient data to compare the different types of RCC in the same kidney with bilateral multifocal or unifocal tumors of the kidney in terms of survival or oncologic survey. Bilateral synchronous renal tumors have been evaluated in many studies [2–4]. Patel et al. [17] more recently found that malignant concordance was 89% among the patients who had bilateral synchronous renal tumors. On the other hand, there is no such data for unilateral synchronous different type of RCC.

In conclusion, we present an additional case of synchronous clear cell and papillary type RCCs in the same kidney. To the best of our knowledge, this is the 12th case of the literature that had these 2 different subtypes of RCC synchronously arising within a single kidney. Large numbers of studies are needed to make a comparison and comment on course of disease because this is only a single case without any controls or comparisons, so clinical implication is limited. However, synchronous 2 different types of RCC in same kidney should not change the management approach.

## Abbreviations

AML: Angiomyolipoma
CT: Computed tomography
MRI: Magnetic resonance imaging
PN: Partial nephrectomy
RN: Radical nephrectomy
RR: Relative risk
RCC: Renal cell carcinoma
TSC: Tuberous sclerosis.
